# Immunogenicity of HLA-DR1 Restricted Peptides Derived from *Leishmania major* gp63 Using FVB/N-DR1 Transgenic Mouse Model

**Published:** 2013

**Authors:** H Rezvan

**Affiliations:** Dept. of Laboratory Science, School of Paraveterinary Sciences, Bu-Ali Sina University, Hamadan, Iran

**Keywords:** *Leishmania major*, gp63, IFN-γ, Peptide, Proliferation, FVB/N-DR1 transgenic mice

## Abstract

**Background:**

Leishmaniasis is a worldwide disease prevalent in tropical and sub-tropical countries. In the present study the immunogenicity of three human HLA-DR1 restricted peptides derived from *L. major* gp63 protein was evaluated using FVB/N-DR1 transgenic mouse model.

**Methods:**

The immunity generated by three MHC class II – restricted peptides with the sequence of AARLVRLAAAGAAVT (AAR), AAPLVRLAAAGAAVT (AAP) and SRYDQLVTRVVTHE (ASR) derived from *L. major* gp63 protein were predicted using a web-based software (SYFPEITHI) and tested in FVB/N-DR1 transgenic mice.

**Results:**

Immunization of FVB/N-DR1 transgenic mice with one of the three predicted peptides (AAR) resulted in high levels of Th1-type immune response as well as significant levels of IFN-γ detected by Proliferation assay and ELISA.

**Conclusion:**

The results indicate a high level of immunogenicity for AAR, which can be a potent candidate for peptide vaccine in *Leishmania* infections.

## Introduction

Today, Leishmaniasis is considered as a neglected disease, which is endemic in many countries. However, the geographical distribution of Leishmaniasis is restricted to tropical and temperate regions and the living area of the sandfly in Asia, India, Africa and the Mediterranean (Old World) and Americas (New World) (1).

According to the estimation of WHO, the number of Leishmaniasis cases accounts for 12 million worldwide and a further 367 million is at risk of acquiring the disease. The number of *Leishmania* new cases is 1.5 - 2 million annually (2–5). Moreover, AIDS patients are more sensitive to *Leishmania* infection than healthy individuals as many of such new cases have been reported from AIDS suffering patients. Vaccination is one of the most feasible and cost effective methods for the control of infectious diseases (6). Different studies in Iran and other countries have been carried out to address candidate vaccines for *Leishmania* (7–10). Although conventional vaccines induce sufficient immunity to some pathogens, they are not effective enough against *Leishmania* (11). Therefore, emphasize is now focused on using a single antigenic protein of pathogens in order to induce specific immunity, which is in turn protective against the pathogen. A recombinant subunit vaccine based on the insertion of a 27-amino acid sequence from Omp31 to the N-terminus of *Brucella* enzyme lumazine synthase (BLS) induced protection against *Brocella ovis* similar to that of the Rev.1 vaccine, inducing a strong peptide-specific humoral, Th1 and cytotoxic T-cell responses (12). *Leishmania* gp63 or leishmanolysin is a characterized immunogenic protein of all *Leishmania* species (13). In this study, it was tried to find epitopes, which are potential to be used as vaccine against *Leishmania*.

## Materials and Methods

### Parasite

*Leishmania* parasites, cells and infection *L. major* promastigotes strain M379 were kindly gifted by Dr. Varley, the London School of Hygiene and Tropical Medicine (LSHTM), and cultured in Schneider media (Sigma, US) supplemented with 10% FCS at 25 °C as described by (14).

### Animals

FVB/N-DR1 mice were received from Dr. Altman and bred at The Nottingham Trent University animal house. FVB/N-DR1 F2 mating positive animals were maintained inbred by ensuring they have a common F0 ancestor.

### Peptides

A list of the peptides used in this study is shown in (Table 1). The peptides were selected using web based algorithm SYFPEITHI. Three peptides, which were predicted to bind to HLA-DR1 molecule with high affinity were chosen and purchased from Alta Biosciences.

**Table 1:** Predicted peptides from *L. major* gp63 proteins by Web-based software “SYFPEITI”

Gene	Sequence	Abbreviation	Class II
*L. major* gp63	AARLVRLAAAGAAVT	AAR	
*L. major* gp63	AAPLVRLAAAGAAVT	AAP	HLA-DR1
*L. major* gp63	ASRYDQLVTRVVTHE	ASR	

### Immunizations

Animals were immunized with 100µg of the peptide diluted in PBS and emulsified in 1:1 dilution with incomplete Freund's adjuvant (IFA) (Sigma, US). The vaccination in the tail close to the site of infection led to a stronger induction of immunity in mice (15). Totally, 100 µl of each peptide emulsion was administered subcutaneously at the tail base of animals. Two rounds of immunization with the same peptide were undertaken at seven-day intervals.

### Proliferation assays

#### BM-DC generation for proliferation assay

BM-DC was generated as described by Inaba with modifications (16). Briefly, hind limbs of naïve mice were harvested and all muscles were removed using scalpel and tweezers. After cutting the ends of the bones, bone-marrow was flushed out and cells were collected, centrifuged, re-suspended in 1ml BM-DC media [complete RPMI, 5% FCS (by volume), 1% glutamine (by volume), 20 mM HEPES, 50µM 2-mercaptoethanol, 50 U/ml penicillin/streptomycin, 0.25 µg/ml fungizone] and plated at 1×10^6^ cells per well/ml with 100ng/ml of mGM-CSF. On day 2 and day 4, non-adherent cells were washed out by gently removing 700µl of media from each well, and adding 750µl of fresh media containing GM-CSF in its place. On day 7, BM-DC was replaced with the 10µg/ml peptide of interest or control peptide for 4-6 hours. LPS was then added at 1µg/ml to induce complete maturation. The cells were then incubated overnight at 37° C, 5% CO_2_. The following day, BM-DC were washed in T-cell media [complete RPMI, 10% FCS (by volume), 1% glutamine (by voume), 20 mM HEPES, 50µM 2-mercaptoethanol, 50 U/ml penicillin /streptomycin, 0.25 µg/ml fungizone], re-suspended in 1ml of T cell media and pulsed with 10µg/ml of peptide for 4-6 hours at 37° C, 5% CO_2_. These cells were plated at 5×10^3^ per well in a round bottom 96 well plate. BM-DC was always used at a 1 DC to 10 splenocyte ratio in proliferation assay.

### Splenocyte preparation and re-stimulation with peptide in vitro

Spleens of immunized animals were harvested and the cells flushed out with T cell media. Cells were collected and placed on ice while the spleen was being digested. The remaining spleen tissue was digested using an enzyme cocktail (0.1U/ml DNAase (Sigma) + 1.6 mg/ml collagenase (Sigma) for 1 hour at 37° C. Single cell suspension was prepared and cells were counted and plated in 24 well plates at 2.5 × 10^6^ cells per well in 1 ml of T cell media and 10 µg/ml of the peptide was added to each well. Supernatants were collected for cytokine assay on day 2 and 5. On day 6, cells were used at 5 × 10^4^ cells per well as responders for the proliferation assay.

### Murine CD8+ T cell depletion

Depletions were done on day 6, using CD8 specific dynabeads (Dynabeads Mouse CD8, Dynal) and following the manufacturer instructions. CD8+ cells attached to the beads were depleted using a magnet. The remaining cells were collected, washed once with T cell media and subsequently used for the proliferation assay or plated in a 48 well plate (17). Protocol for CD8+ depletion has been optimized in our laboratory and the remaining cells consisted negligible number of CD8+ T cells.

### Proliferation assay for murine CD4+ T-cells

The cells collected after T cell depletion, were re-suspended in 4 ml of T cell media and counted. These cells were then plated out in 96 well round bottom plate at cell density of 5×10^4^ cells per well. Peptide pulsed BM-DC were used as antigen presenting cells in all the experiments. Responder cells were co-cultured with BM-DC either pulsed with the relevant peptide, and irrelevant peptide or no peptide in some experiments. Peptide pulsed BM-DC were added to the wells at a density of 5×10^3^ cells per well. To ascertain the MHC restriction of the response, a MHC blocking anti-HLA-DR antibody was added to the relevant wells. A matched isotype antibody was also used as control in these experiments. Each culture was performed in triplicates or quadruplicates for approximately 60 hours. Tritiated thymidine (Amersham, UK) was added at a final concentration of 0.037MBq/ml 16 to 18 hours prior to harvesting. Cells were harvested using a 96-well harvester (Packard) onto a 96-well UniFilters GF/C plate (Packard) and the plate was left to dry for 1 hour in a drying cabinet. 40 µl of scintillation fluid (Microscint 0, Packard) was added to each of the filter wells. Filters were counted on a Top-Count scintillation counter (Packard).

### Cytokine Assays (IFN-γ & IL-4)

Splenocytes were prepared as outlined above. 1 ml of the cell culture supernatant on days 2 and/or 5 was collected and stored at -20C until required. Cytokine analysis for IFN-γ and IL-4 using the ELISA kits (R&D Systems, Abingdon, UK) was performed according to the manufacturer's protocols.

### Peptide predictio

The web-based software “SYFPEITHI” was used to predict imunogenic MHC class II - restricted peptides derived from *Leishmania major* gp63 protein (gene bank re Y00647) with high affinity to human HLA-DR1 molecules.

## Results

### BM-DC cells

To assess the phenotyping of DCs used in prolifration assay, 5 × 10^5^ per tube DCs were harvested for FACS analysis. Cells were washed twice in PBS + 0.1%BSA + 0.02%NaN_3_. Rat anti-mouse CD80, Macrophage/Monocyte marker (F4/80), DEC205, I-A (murine class II) and CD45, and hamster anti-mouse CD11c monoclonal antibodies were added. Appropriate isotype controls were used in each experiment.

**Fig. 1:**
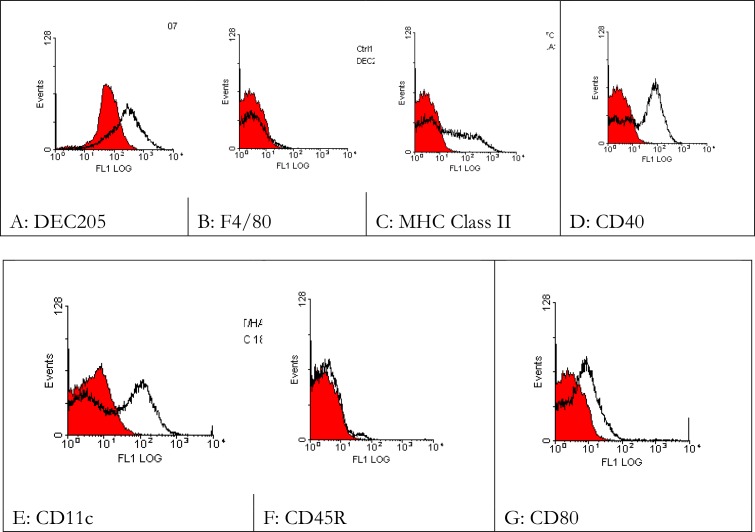
DC phenotypic analysis

The cells were incubated on ice for 30 minutes with primary antibodies. Cells were then washed twice in PBS + 0.1%BSA + 0.02%NaN_3_ and incubated for 30 minutes on ice with FITC coupled goat anti-rat IgG or goat anti-hamster IgG as secondary antibodies as appropriate. Finally the cells were washed in PBS + 0.1%BSA + 0.02%NaN_3_ and resuspended in 500µl of sheath fluid, and analysed by FACS. Results showed expression of markers in the cultured DCs (Fig. 1).

Bone marrow cells obtained from BALB/c mice were cultured in presence of GM-CSF for 6 days with gentle wash every two days. On day 6 the DCs were treated with relevant and irrelevant peptides (10-15µg/ml) and after 4-6h they were pulsed with LPS 1µg/ml to mature. On day 7 the cells were split into a number of groups stained with Abs and phenotyped by FACS analysis. Red line: control; Black line: test.

### Peptide vaccination in FVB/N-DR1 transgenic mice

**Fig. 2:**
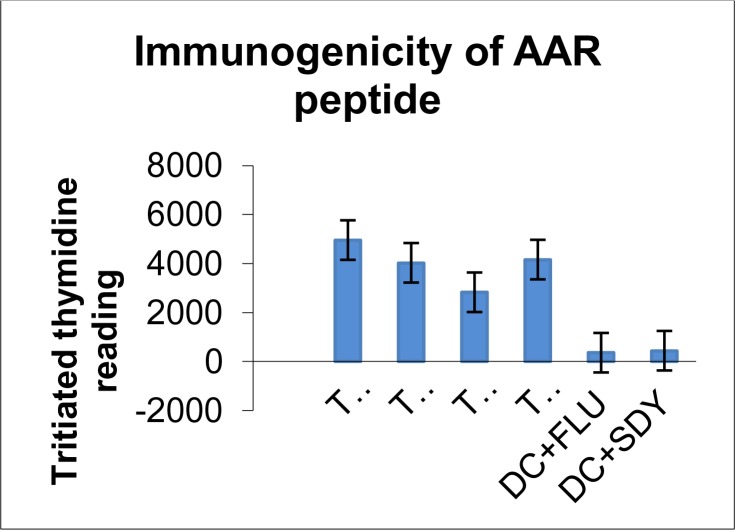
Immunogenicity of AAR peptide in proliferation assay: 8 weeks female FVB/N-DR1 animals were immunised with 100µg of the peptide diluted in PBS and emulsified in 1:1 dilution with incomplete Freund's adjuvant (IFA) (Sigma). 100µl of the peptide emulsion was administered at the tail base of animals two times at seven-day intervals. Proliferation assay was performed to assess the immunogenicity of the peptides. P-Value for the level of proliferation between test and control was 0.022

Peptides from gp63 proteins of *L. major* class II molecules were selected by using the prediction web-based software “SYFPEITHI” and their immunogenicity was determined in FVB/N-DR1 transgenic mice. Three peptides named AAR, AAP and ASR were tested in FVB/N-DR1 mice. Only AAR showed immunogenicity compared with the irrelevant peptides (Fig. 2).

### Cytokine Assays (IFN-γ & IL-4)

**Fig. 3:**
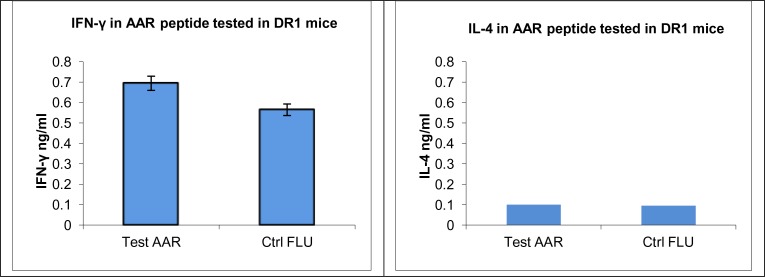
IFN-γ and IL-4 production by splenocytes from FVB/N-DR1 mice immunised with AAR: **S**upernatants collected from splenocytes cultured with DCs pulsed with AAR and an irrelevant peptide (FLU) on the day 2 and 5 were stored at - 20 until required. IFN-γ and IL-4 were measured using commercial kits according to manufacture's instruction (see materials and methods). *P*-value for the level of IFN-γ between test and control was 0.003

The results were confirmed by determining the levels of IFN-γ and IL-4. The amount of IFN-γ in samples collected from splenocytes of the immunized mice cultured with DCs pulsed with relevant peptide (Test) was significantly higher than those pulsed with irrelevant peptide (control). No significant IL-4 (a key cytokine in activation of Th2 pathway) levels were detected (Fig. 3).

## Discussion

Peptide immunization is a new vaccination approach that has not yet fully investigated in *Leishmania* vaccination. Gp63, a *Leishmania* antigen, has been postulated as promising candidates for *Leishmania* peptide-subunit vaccine. In a study by Spitzer a 16-mer synthetic peptide with the sequence of YDQLVTRVVTHEMAHA derived from *L. major* gp63, induced a detectable immunity in BALB/c mice (18). On the other hand, there are many studies, including our own, which have demonstrated immunogenicity of gp63 proteins in *Leishmania*. Therefore, it is appropriate to identify MHC calss II restricted epitopes that can be used as a vaccine to *Leishmania* either alone or in combination with other immunogenic or therapeutic agents. This study is to reports the identification of immunogenic MHC class II restricted epitopes from *Leishmania* gp63 protein using FVB/N-DR1 transgenic mice. In order to identify immunogenic epitopes, which are presented through MHC class II molecules, the web-based software “SYFPEITHI” was used to predict the immunogenic peptides (19–20). The immunogenicity of the predicted peptides was determined by using a number of immunological tests. Due to the ethical difficulties associated with studies on human subjects, FVB/N-DR1 mice were used to determine the immunogenicity of the peptides predicted for human HLA-DR molecules. Transgenic mice have been described as a powerful model to study human immune responses (20, 21–25). Using *L. major* gp63 sequences, three of fifteen mer peptides named AAR, AAP and ASR with the sequence of AARLVRLAAAGAAVT, AAPLVRLAAAGAAVT, ASRYDQLVTRVVTHE were predicted to have affinity to HLA-DR1 molecules and were tested for immunogenicity in HLA-DR1transgenic mice. One peptide (AAR) induced immunity in the immunized mice by inducing proliferation in DCs pulsed the relevant peptides. IFN-γ secreted by T cells has been shown to be essential for the development of Th1 responses and it has been used as a marker for the existence of Th1 immune responses, while IL-4 on the other hand indicates the bias immunity towards the Th2 pathway (26). The level of IFN-γ produced by splenocytes from mice immunized with immunogenic *Leishmania* gp63 peptides (AAR) cultured with DCs pulsed with relevant peptides confirmed its role in the activation of Th1 pathway. Immunization with the other two peptides failed to produce a significant level of IFN-γ. The lack of IL-4 secretion may indicate down regulation or the absence of Th2 responses in this model.

## Conclusion

*Leishmania* gp63 is a potent protein for developing a peptide subunit vaccine for *Leishmania* and using the computer-based prediction is feasible method for prediction of human MHC class II epitopes to help pave the way for future studies on finding potent epitopes to be used as a vaccine against *Leishmania*. Moreover, enhancing the immunogenicity of peptides should be pointed out in future studies to find better vaccines.
